# Risk Factors for the Acquisition of *Enterococcus faecium* Infection and Mortality in Patients with Enterococcal Bacteremia: A 5-Year Retrospective Analysis in a Tertiary Care University Hospital

**DOI:** 10.3390/antibiotics10010064

**Published:** 2021-01-11

**Authors:** Atsushi Uda, Katsumi Shigemura, Koichi Kitagawa, Kayo Osawa, Kenichiro Onuma, Yonmin Yan, Tatsuya Nishioka, Masato Fujisawa, Ikuko Yano, Takayuki Miyara

**Affiliations:** 1 Department of Infection Control and Prevention, Kobe University Hospital, Kobe 650-0017, Japan; katsumi@med.kobe-u.ac.jp (K.S.); onumak@med.kobe-u.ac.jp (K.O.); miyarat@med.kobe-u.ac.jp (T.M.); 2 Department of Pharmacy, Kobe University Hospital, Kobe 650-0017, Japan; tnishi@med.kobe-u.ac.jp (T.N.); iyano@med.kobe-u.ac.jp (I.Y.); 3 Division of Infectious Diseases, Department of Public Health, Kobe University Graduate School of Health Sciences, Kobe 654-0142, Japan; ko1.kitgwa@gmail.com; 4 Division of Urology, Kobe University Graduate School of Medicine, Kobe 650-0017, Japan; yym1112@gmail.com (Y.Y.); masato@med.kobe-u.ac.jp (M.F.); 5 Division of Advanced Medical Science, Kobe University Graduate School of Science, Technology and Innovation, Kobe 657-8501, Japan; 6 Department of Medical Technology, Kobe Tokiwa University, Kobe 653-0838, Japan; osawak@kobe-u.ac.jp; 7 Department of Clinical Laboratory, Kobe University Hospital, Kobe 650-0017, Japan

**Keywords:** enterococcal, bacteremia, epidemiology, risk factors, mortality, antimicrobial stewardship

## Abstract

The incidence of bacteremia caused by *Enterococcus faecium*, which is highly resistant to multiple antibiotics, is increasing in Japan. However, risk factors for the acquisition of *E. faecium* infection and mortality due to enterococcal bacteremia are not well known. We compared demographic, microbiological, and clinical characteristics using a Cox regression model and univariate analysis. We performed a multivariate analysis to identify risk factors for patients treated between 2014 and 2018. Among 186 patients with enterococcal bacteremia, two groups included in the Kaplan–Meier analysis (*E. faecalis* (n = 88) and *E. faecium* (n = 94)) showed poor overall survival in the *E. faecium* group (HR: 1.92; 95% confidence interval: 1.01–3.66; *p* = 0.048). The median daily antibiotic cost per patient in the *E. faecium* group was significantly higher than that in the *E. faecalis* group ($23 ($13–$34) vs. $34 ($22–$58), *p* < 0.001). *E. faecium* strains were more frequently identified with previous use of antipseudomonal penicillins (OR = 4.04, *p* < 0.001) and carbapenems (OR = 3.33, *p* = 0.003). Bacteremia from an unknown source (OR = 2.79, *p* = 0.025) and acute kidney injury (OR = 4.51, *p* = 0.004) were associated with higher risks of 30-day mortality in patients with enterococcal bacteremia. Therefore, clinicians should provide improved medical management, with support from specialized teams such as those assisting antimicrobial stewardship programs.

## 1. Introduction

*Enterococcus* species are Gram-positive, facultative anaerobic cocci that constitute the normal bacterial flora in human and animal intestines. Enterococcal bacteremia is associated with a high mortality rate and prolonged hospitalization [1,2,3]. *Enterococcus faecalis*, followed by *E. faecium* are the most frequent *Enterococcal* species that cause bacteremia [4]. *Enterococcus* species are intrinsically cephalosporins-resistant, which inhibit bacterial cell wall synthesis. Primarily, infections caused by vancomycin-resistant enterococci are associated with higher mortality and are a major problem in the United States and Western countries [3,5,6]. However, the prevalence of vancomycin-resistant *Enterococcus* infections in Japan is markedly lower than that in other countries [4,7,8]. In contrast, most *E. faecalis* are susceptible to penicillins, although *E. faecium* tends to have resistance to some antimicrobial agents, including penicillins, aminoglycosides, and carbapenems [4]. The hospital cost and mortality for patients with multidrug-resistant pathogens were higher than those with antimicrobial-susceptible pathogens [9,10,11]. Many studies have reported that previous use of broad-spectrum antibiotics is a risk factor for acquiring the multidrug-resistant pathogens [12,13,14]; however, few studies have been done to identify the correlation between the previous antibiotic exposure and the acquisition of *E. faecium* strains [15]. In the United States and Western countries, the enterococcal isolates of *E. faecalis* (80–90%) and *E. faecium* (5–20%) [8,16] are considerably different than those in Japan (*E. faecium* strains account for 40%) [4]. Although there are many studies on enterococcal bacteremia, the clinical outcomes, epidemiological features, and risk factors for nosocomial infection produced different results depending on the country, hospitalization ward, or patient characteristics. Only few studies have described the situation in Japan [17,18], and the conclusions are inconsistent. Therefore, we aimed to investigate the clinico-epidemiological features and risk factors predisposing to the acquisition of *E. faecium* strains and mortality due to nosocomial enterococcal bacteremia.

## 2. Results

### 2.1. Patient Data

**Figure 1:**
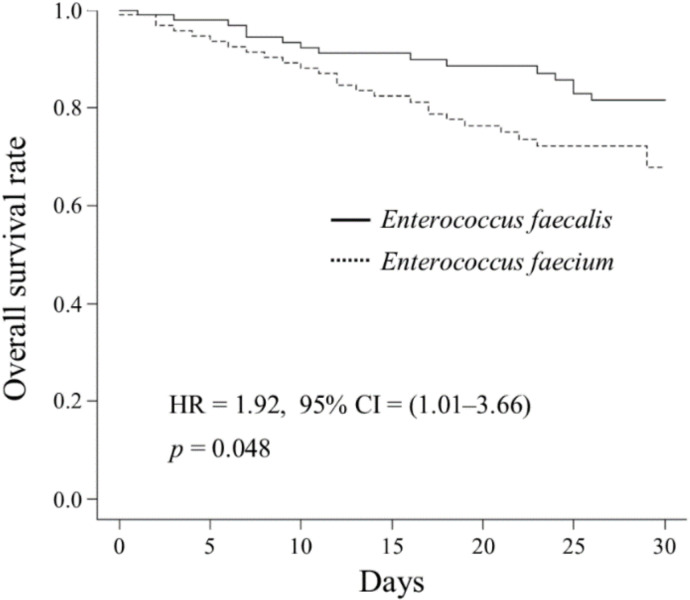
Kaplan–Meier survival curves of patients with *E. faecalis* and *E. faecium* bacteremia.

During the 5-year study period (2014–2018), 186 patients had bacteremia caused by *Enterococcus* species. The most common *Enterococcus* species were *E. faecium* (n = 94, 51%), followed by *E. faecalis* (n = 88, 47%), *E. avium* (n = 1, 0.5%), *E. casseliflavus* (n = 1, 0.5%), *E. raffinosus* (n = 1, 0.5%), and *E. gallinarum* (n = 1, 0.5%). As few patients had bacteremia caused by *E. avium*, *E. casseliflavus*, *E. raffinosus*, and *E. gallinarum*, only the clinical characteristics of bacteremia with *E. faecalis* and *E. faecium* were further investigated among two subgroups. After excluding four cases of bacteremia caused by strains other than *E. faecalis* or *E. faecium*, 182 patients were eligible for inclusion in this study. The survival rates for both study groups are shown in the Kaplan–Meier curves in Figure 1, showing a significant decrease in overall survival rates among the patients with *E. faecium* bacteremia (hazard ratio (HR): 1.92; 95% confidence interval (CI): 1.01–3.66; *p* = 0.048).

### 2.2. Demographic and Clinical Characteristics

The demographic and clinical characteristics of the study participants are shown in Table 1. There was no significant difference between groups regarding sex, age, hospitalization ward, length of hospitalization before the onset of bacteremia, quick Sequential Organ Failure Assessment (qSOFA) score ≥ 2, use of invasive devices, or surgical history. The coexistence of hepatobiliary (*p* = 0.005) and hematologic (*p* = 0.027) tumors were more frequently observed in patients with *E. faecium* bacteremia. The most common primary source of infection arose from an insertion of a central venous catheter (n = 41, 23%), followed by cholecystocholangitis (n = 38, 21%), urinary tract infection (n = 18, 9.9%), and intra-abdominal infection (n = 12, 6.6%). *E. faecium* bacteremia originated more frequently from cholecystocholangitis (*p* < 0.001) and febrile neutropenia (*p* = 0.015) than in the *E. faecalis* group. However, the *E. faecalis* group had an unknown source of infection more frequently (*p* = 0.041). The incidence of urinary tract infections was lower in *the E. faecium* group than in the *E. faecalis* group (*p* = 0.017). A history of antibiotic therapy with antipseudomonal penicillins (*p* < 0.001) and carbapenems (*p* < 0.001) was more frequently observed in the *E. faecium* group. Based on the analysis by a logistic regression model, preexisting hematologic tumors (adjusted OR = 7.85, *p* = 0.004), cholecystocholangitis (adjusted OR = 5.21, *p* = 0.001), and previous use of both antipseudomonal penicillins (adjusted OR = 4.04, *p* < 0.001) and carbapenems (adjusted OR = 3.33, *p* = 0.003) were independent risk factors for the acquisition of an *E. faecium* infection. 

### 2.3. Microbiological Data

Table 2 shows the microbiological characteristics of patients with enterococcal bacteremia. There was no significant intergroup difference in polymicrobial cultures; all isolates of *E. faecalis* and 15% (14/94) strains of *E. faecium* were susceptible to ampicillin. We found no vancomycin-resistant isolates among the enterococci. No imipenem-resistant *E. faecalis* strain was isolated; however, all *E. faecium* isolates were imipenem-resistant. Susceptibility to levofloxacin was detected in 91% (80/88) and 12% (11/94) of *E. faecalis* and *E. faecium* isolates, respectively.

### 2.4. Clinical Management and Outcomes

Table 3 shows the clinical management and outcomes of patients with bacteremia caused by *E. faecalis* and *E. faecium* infections. The rate of source control with drainage in the *E. faecium* group was ~1.7 times higher than that in the *E. faecalis* group, although the difference was not significant (*p* = 0.15). Non-antipseudomonal penicillins (n = 55, 63%) was the most common antibiotic used for *E. faecalis* bacteremia, whereas vancomycin (n = 74, 79%) was most frequently prescribed in *E. faecium* bacteremia. Compared with *the E. faecium* group, the *E. faecalis* group more frequently received penicillins (*p* < 0.001) and aminoglycosides (*p* = 0.031). Vancomycin (*p* < 0.001) and other anti-methicillin-resistant *Staphylococcus aureus* (MRSA) agents (*p* = 0.028) were more frequently administered in the *E. faecium* group. Non-antipseudomonal penicillins (*p* = 0.022) and quinolones (*p* = 0.022) were prescribed for longer durations in the *E. faecalis* group, whereas the median duration of vancomycin use was longer in the *E. faecium* group (*p* < 0.001). When vancomycin treatment took more than three days, all patients underwent therapeutic drug monitoring. The *E. faecalis* group showed a shorter median duration to the commencement of initial antibiotic therapy against enterococci (*p* = 0.049), although there was no significant intergroup difference in the total duration of antibiotic treatment (*p* = 0.99). The median daily antibiotic cost per patient in the *E. faecium* group was significantly higher than that in the *E. faecalis* group ($23 [$13–$34] vs. $34 [$22–$58], *p* < 0.001). Patients in the *E. faecium* group more frequently attained a vancomycin median serum trough concentration ≥20 mg/L (*p* = 0.007) than in the *E. faecalis* group. Acute kidney injury (AKI) was observed in both groups but was more frequent in *the E. faecium* group (*p* = 0.02). The clinical outcome for patients with enterococcal bacteremia was analyzed based on the length of hospitalization; however, no significant between-group difference (*p* = 0.34) was observed.

In this study cohort, the overall 30-day mortality rate was 23% (41/182). Table 4 shows the risk factors associated with the overall 30-day mortality in patients with enterococcal bacteremia. Patient groups with variables such as admission to an intensive care unit, an unknown source of infection, qSOFA score ≥ 2, previous immunosuppressive and corticosteroid treatment, or encountered AKI were associated more frequently with 30-day death. These variables were included in the multivariate logistic regression analysis revealing the following independent risk factors for mortality: unknown source of infection (OR = 2.79, *p* = 0.025), qSOFA score ≥ 2 (OR = 2.96, *p* = 0.024), previous corticosteroid treatment (OR = 2.84, *p* = 0.034), and AKI (OR = 4.51, *p* = 0.004).

## 3. Discussion

This observational retrospective study analyzed the epidemiological and clinical outcomes of enterococcal bacteremia and evaluated the risk factors for the acquisition of *E. faecium* and *E. faecalis* infection and mortality in enterococcal bacteremia. We found a significant increase in *E. faecium* bacteremia among patients with enterococcal bacteremia, especially those who were previously treated with antipseudomonal penicillins and carbapenems. We also found that severely ill patients, those with an unknown source of infection, and AKI during treatment conferred higher risks of mortality in enterococcal bacteremia. The results of our analyses suggest the need for greater efforts to provide accurate medical treatment, including appropriate antimicrobial use in patients with enterococcal bacteremia.

As a leading cause of nosocomial bacteremia, enterococci have become more prevalent worldwide. In particular, the spread of vancomycin-resistant *Enterococcus* has become a major public health problem in the United States and in Western Europe [3,5,6]. However, we found no vancomycin-resistant strains of enterococci in our hospital during the 5-year study period. *E. faecalis* and *E. faecium* are two major *Enterococcus* species that can cause various complicated infectious diseases [3,4,16] and were the most common strains in our cohort. In agreement with the results of an earlier study [19], we observed significantly lower survival rates with *E. faecium* than with *E. faecalis*, as determined using the Kaplan–Meier survival curves. Furthermore, the *E. faecium* group showed a higher daily antibiotic cost than the *E. faecalis* group. Infections with drug-resistant pathogens are typically associated with increased hospitalization costs [9]. De-escalation therapy to narrower spectrum antibiotics is a cost-saving strategy [20,21]. After bacterial identification, antibiotics should be changed, if needed, to administer appropriate targeted antibiotic therapy in accordance with bacterial culture and susceptibility data. In general, patients infected with enterococcal bacteremia are older and more likely to develop renal failure; thus, less toxic regimens such as penicillins may be preferred. In this study, because all the *E. faecalis* remained susceptible to penicillins, the *E. faecalis* group received narrower spectrum antibiotics, such as non-antipseudomonal penicillins. Whereas the *E. faecium* group, which had a higher resistance to penicillins, was most frequently prescribed vancomycin. Non-antipseudomonal penicillins, which constitute the most prescribed antibiotics in the *E. faecalis* group, were of a lower price in Japan than anti-MRSA agents [22], which resulted in a lower median daily antibiotic cost in the *E. faecalis* group. We demonstrated that *E. faecium* bacteremia caused more serious problems regarding therapeutic outcomes.

Previous studies have focused on risk factors for the acquisition of *E. faecium* infections among various ethnic groups [15,23], but little is known about the risk factors in Japan [17]. In this study, multivariate analysis revealed that the risk factors for bacteremia due to *E. faecium* included preexisting illness (hematologic tumor), source of infection (cholecystocholangitis), and previous use of broad-spectrum antibiotics (antipseudomonal penicillins and carbapenems). For patients with nosocomial or intra-abdominal infections, broad-spectrum antibiotics were often prescribed to treat anaerobic bacteria and Gram-negative bacteria such as *Pseudomonas aeruginosa*. In a previous report, carbapenems were reported as the only independent risk factor associated with *E. faecium* bloodstream infections [15]. However, this study is the first to characterize antipseudomonal penicillins as a predictive risk factor for the acquisition of *E. faecium*. In Japanese national and public university hospitals, the consumption of antipseudomonal penicillins increased five times from 2008 to 2015 [24], and the rates of *E. faecium* among enterococcal bacteremia patients increased from 36% to 43% in the same period [4]. The higher rates of *E. faecium* isolation might correlate with the increased use of antipseudomonal penicillins in Japan. Broad-spectrum antibiotics might destroy the normal anaerobic flora of the gastrointestinal tract by selective elimination of enterococci due to the bactericidal activity against these organisms, which might subsequently induce infectious diseases. A case-control study revealed that broad-spectrum antibiotic therapy, including antipseudomonal penicillins, was a risk factor for *P. aeruginosa* resistance among hospitalized patients [25]. The Infectious Diseases Society of America guidelines recommend the implementation of antibiotic stewardship programs to restrict the prevalence of antimicrobial-resistant pathogens [26]. Overuse of broad-spectrum antibiotics leads to the selective growth of resistant bacteria; thus, one of the aims of antimicrobial stewardship is to promote appropriate antibiotic use. Our hospital has practiced antimicrobial stewardship since 2010 to optimize antibiotic usage [27,28,29]; however, the consumption of antipseudomonal penicillins significantly increased between 2009 and 2016 [27]. Thus, narrower spectrum antibiotics should be prescribed to avoid the development and prevalence of bacterial resistance as much as possible with consideration to preexisting infectious diseases and patient conditions even before the onset of bacteremia.

Empirical antibiotic therapy is often commenced before pathogen identification and without susceptibility data. *E. faecium* is typically resistant to penicillins [4]; thus, when enterococcal infection is suspected, initial empiric therapy often requires the prescription of anti-MRSA agents, especially vancomycin. We found that the *E. faecium* group showed a longer duration of vancomycin use, higher vancomycin trough levels, and higher rates of AKI. Higher vancomycin trough levels have been previously identified as a risk factor for nephrotoxicity [30], and thus, the longer duration of vancomycin use in the *E. faecium* group might have elevated the vancomycin trough levels leading to renal injury. Furthermore, glycopeptide use is associated with higher mortality in patients with *E. faecalis* bacteremia [31]. These findings suggest that vancomycin doses need to be considered carefully for patients with enterococcal bacteremia to prevent AKI.

Risk factors for mortality due to enterococcal bloodstream infections may include malignancy, admission to the intensive care unit, severity of illness, and high-level resistance to ampicillin and ciprofloxacin [1,15,18,32,33]. In this study, we identified AKI, unknown source of infection, previous corticosteroid treatment, and qSOFA score ≥ 2 as independent risk factors associated with mortality due to enterococcal bacteremia. These findings may be clinically plausible because impairment of kidney function and consequent multiorgan dysfunction syndrome, which likely leads to mortality, occurs in critically ill patients [34,35]. Moreover, severely ill patients, such as those with collagenase disease, nephrotic syndrome, and advanced cancer, receive corticosteroid therapy. However, corticosteroids not only decrease inflammation but also have side effects, including a reduction in the activity of the immune system, as well as hyperglycemia. A previous study reported that an unknown focus of bacteremia was associated with inappropriate antibiotic therapy and poor clinical outcome [36]. These findings suggest that the risk factors associated with mortality provide useful information for clinicians to avoid treatment failure, thereby enabling appropriate medical therapy after the onset of bacteremia. Specialized personnel, such as antimicrobial stewardship teams, can support clinical management through appropriate antibiotic use and early diagnosis.

This study has some limitations. First, we conducted a retrospective study in a single university hospital, and the data were gathered by reviewing electronic medical records, relying on other investigators for data collection; hence, a measurement bias could not be ignored. Second, this study investigated only *E. faecalis* and *E. faecium* bacteremia. To evaluate the characteristics of enterococci, we must assess the clinical outcomes of patients with bacteremia caused by *Enterococcus* species, including *E. avium*, *E. casseliflavus*, and *E. raffinosus*, other than *E. faecalis* and *E. faecium*.

## 4. Materials and Methods 

### 4.1. Setting and Patients

We conducted an observational retrospective study between 1 January 2014, and 31 December 2018, at Kobe University Hospital. We defined nosocomial enterococcal bacteremia as positive blood cultures obtained after 48 h of hospitalization. We investigated all adult patients (age > 18 years) with only the first episode of at least one positive blood culture for *Enterococcus* species. In patients with two or more blood cultures for the same organism, only one was included in the analysis. Patient data were obtained from electronic medical records.

### 4.2. Definitions

The demographic information included age and sex. The clinical and microbiological data for each case were carefully reviewed. The hospitalization ward, comorbidities, the source of infection, qSOFA score, and use of invasive devices were evaluated on the day of bacteremia onset. Data pertaining to recent surgery, immunosuppressive treatment, and previous antibiotic therapy within 30 days prior to the first positive blood culture were collected. The polymicrobial culture was defined as the isolation of more than one organism, excluding contaminated pathogens, which were defined if the following pathogens (coagulase-negative staphylococci, *Bacillus* species, *Corynebacterium* species, *Propionibacterium* species, Viridans-group *streptococci*, and *Micrococcus* species) were detected in one of two or more blood culture sets on the same day. Antibiotic therapy against enterococci was defined as the prescription of antibiotics to which the isolated enterococci were susceptible to bacteremia. Source control with surgical drainage, time to the initiation of antibiotic therapy against enterococci, and total duration of antibiotic treatment was reviewed from the onset of bacteremia. Daily antimicrobial cost during antibiotic therapy for enterococcal bacteremia was calculated by multiplying the drug prices per dose by the total number of given doses and dividing the product by the total number of days of antibiotic therapy. All costs are shown in US dollars ($; exchange rate, 1 $ = 104.30 yen in 1 December 2020). We counted the number of patients whose vancomycin median serum trough concentration was ≥20 mg/L. AKI was defined as an absolute increase in the serum creatinine level to >0.3 mg/dL or a >1.5-fold increase from the baseline value within 7 days. We defined the 30-day mortality as death due to any cause within 30 days after the onset of bacteremia.

### 4.3. Identification and Antibiotic Susceptibility Testing

The BACTEC FX system (Becton and Dickinson, Tokyo, Japan) was used to process the blood cultures. Enterococci were isolated according to standard microbiological procedures. The isolates were identified using matrix-assisted laser desorption/ionization time-of-flight mass spectrometry (MALDI-TOF MS; Bruker, Tokyo, Japan). The minimum inhibitory concentrations of ampicillin, vancomycin, imipenem, and levofloxacin were determined by a broth microdilution method using Microscan Walk Away 96plus (Beckman Coulter, Tokyo, Japan) and interpreted according to the breakpoints proposed by the Clinical and Laboratory Standards Institute guidelines. *Staphylococcus aureus* (ATCC 29213, VA, USA) was used as the quality control strain.

### 4.4. Statistical Analysis

Continuous variables are expressed as medians with interquartile ranges (IQRs) and categorical variables as frequency counts with percentages. Continuous variables were compared using the Mann–Whitney *U* test. Categorical variables were compared using the Chi-square test. Multivariate conditional logistic regression analysis of factors that were potentially associated with *E. faecium* acquisition and mortality included clinically important variables of the statistically significant variables in the univariate analysis. We assessed the in-hospital mortality by using Kaplan–Meier analysis and estimated HR and 95% CI using multivariate Cox proportional hazard regression models. All statistical analyses were performed using the statistical software EZR (Saitama Medical Center, Jichi Medical University, Saitama, Japan). 

### 4.5. Ethics Approval

This study was approved by the Kobe University Graduate School of Health Sciences Institutional Review Board (approval no. 472-6) and was performed in accordance with the ethical standards of the Institutional Research Committee and the tenets of the Declaration of Helsinki (1964).

## 5. Conclusions

In summary, we conducted an observational retrospective study to compare the clinical features and outcomes of bacteremia caused by *E. faecalis* and *E. faecium.* Multivariate analysis was used to identify the risk factors for the acquisition of *E. faecium* bacteremia and mortality due to enterococcal bacteremia. This study demonstrated that the *E. faecium* group had a shorter survival period and higher antimicrobial cost than the *E. faecalis* group. *E. faecium* bacteremia occurred more frequently among patients treated with broad-spectrum antibiotics, especially patients with hematologic tumors and cholecystocholangitis. Furthermore, we identified that severe illness, which tends to be associated with a worse renal function without an infectious focus, was an independent risk factor for mortality due to enterococcal bacteremia. These findings suggest that clinicians should provide a rational treatment strategy supported by specialized teams, such as those assisting antimicrobial stewardship programs, even before the onset of bacteremia in hospitalized patients.

## Figures and Tables

**Table 1:** Intergroup comparison of demographic and clinical characteristics of patients with enterococcal bacteremia and risk factors for the acquisition of *E. faecalis* and *E. faecium.*

	*E. faecalis* (n = 88)	*E. faecium* (n = 94)	*p*	Adjusted OR (95% CI)	*p*
Age (years), median (IQR)	73.5 (66–80)	72 (65–75)	0.073		
Male sex, n (%)	53 (60)	54 (58)	0.82		
Hospitalization ward, n (%)					
Medical ward	24 (27)	30 (32)	0.60		
Surgical ward	41 (47)	33 (35)	0.15		
Intensive Care Unit	23 (26)	31 (33)	0.40		
Comorbidities, n (%)					
Chronic renal failure	35 (40)	32 (34)	0.52		
Dialysis	8 (9.1)	10 (11)	0.9		
Diabetes mellitus	21 (24)	18 (19)	0.55		
Cardiovascular disease	23 (26)	12 (13)	0.058		
Previous cardiac valve replacement	11 (13)	9 (9.6)	0.69		
Coronary artery bypass grafting	3 (3.4)	3 (3.2)	0.9		
Hepatobiliary tumor	5 (5.7)	20 (21)	0.005	3.01 (0.87–10.5)	0.083
Other solid tumors	15 (17)	13 (14)	0.69		
Hematologic tumor	3 (3.4)	13 (14)	0.027	7.85 (1.96–31.4)	0.004
Solid organ transplant recipient	1 (1.1)	1 (1.1)	1		
Bone marrow transplant recipient	1 (1.1)	2 (2.1)	1		
Neutropenia	0 (0.0)	9 (9.6)	0.008		
Hepatobiliary disease	6 (6.8)	3 (3.2)	0.43		
Collagen disease	1 (1.1)	10 (11)	0.018	8.41 (0.91–77.7)	0.061
Source of infections, n (%)					
Central venous catheter	18 (21)	23 (25)	0.64		
Cholecystocholangitis	8 (9.1)	30 (32)	<0.001	5.21 (1.89–14.3)	0.001
Urinary tract infection	14 (16)	4 (4.3)	0.017		
Intra-abdominal infection	3 (3.4)	9 (9.6)	0.17		
Febrile neutropenia	0 (0.0)	8 (8.5)	0.015		
Infectious endocarditis	4 (4.6)	0 (0.0)	0.11		
Wound infection	2 (2.3)	1 (1.1)	0.95		
Unknown	24 (27)	14 (15)	0.041		
Others	6 (6.8)	2 (2.1)	0.24		
Hospital stay before the onset of bacteremia (days), median (IQR)	23.5 (8–56.5)	31 (13.3–75.8)	0.13		
qSOFA score ≥ 2, n (%)	27 (31)	29 (31)	1		
Recent surgery, n (%)	32 (36)	31 (33)	0.75		
Invasive devices, n (%)					
Central intravenous catheter	39 (44)	50 (53)	0.29		
Urinary catheter	43 (49)	39 (42)	0.40		
Immunosuppression (within 30 days), n (%)					
Immunosuppressive treatment	2 (2.3)	9 (9.6)	0.079		
Corticosteroid treatment	13 (15)	26 (28)	0.053		
Chemotherapy	5 (5.7)	13 (14)	0.11		
Previous antibiotic therapy (within 30 days)					
Non-antipseudomonal penicillins					
Number of patients (%)	16 (18)	27 (29)	0.13		
Duration of use, median (IQR)	6 (3–9)	5 (2.5–8.5)	0.68		
Antipseudomonal penicillins					
Number of patients (%)	14 (16)	42 (45)	<0.001	4.04 (1.81–9.0)	<0.001
Duration of use, median (IQR)	7 (4.3–9.5)	6 (4–8)	0.89		
Cephalosporins					
Number of patients (%)	51 (58)	53 (56)	0.95		
Duration of use, median (IQR)	5 (3–7)	5 (2–10)	1		
Carbapenems					
Number of patients (%)	16 (18)	42 (45)	<0.001	3.33 (1.51–7.36)	0.003
Duration of use, median (IQR)	7 (3.8–9)	6.5 (4.3–10.8)	0.24		
Quinolones					
Number of patients (%)	9 (10)	19 (20)	0.097		
Duration of use, median (IQR)	4 (4–6)	7 (4–9)	0.69		
Aminoglycosides					
Number of patients (%)	0 (0.0)	3 (3.2)	0.27		
Duration of use, median (IQR)	0 (0–0)	3 (2.5–3.5)	<0.001		
Anti-MRSA agents (VCM)					
Number of patients (%)	15 (17)	28 (30)	0.065		
Duration of use, median (IQR)	5 (2.5–9.5)	4 (2–5.3)	0.26		
Anti-MRSA agents (DAP, LZD)					
Number of patients (%)	6 (6.8)	10 (11)	0.52		
Duration of use, median (IQR)	3.5 (2.3–7)	2.5 (1.3–6)	0.51		

IQR: interquartile range, qSOFA: quick Sequential Organ Failure Assessment, MRSA: methicillin-resistant *Staphylococcus aureus*, VCM: vancomycin, DAP: daptomycin, LZD: linezolid.

**Table 2:** Microbiological characteristics of enterococcal bacteremia.

	*E. faecalis* (n = 88)	*E. faecium* (n = 94)	*p*
Polymicrobial culture, n (%)	22 (25)	29 (31)	0.48
Antibiotic susceptibility, n (%)			
Ampicillin	88 (100)	14 (15)	<0.001
Vancomycin	88 (100)	94 (100)	0.66
Imipenem	88 (100)	0 (0.0)	<0.001
Levofloxacin	80 (91)	11 (12)	<0.001

**Table 3:** Clinical treatments and outcomes of patients with enterococcal bacteremia.

	*E. faecalis* (n = 88)	*E. faecium* (n = 94)	*p*
Source control with drainage, n (%)	13 (15)	23 (25)	0.15
Antibiotic therapy against enterococci			
Non–antipseudomonal penicillins			
Number of patients (%)	55 (63)	4 (4.3)	<0.001
Duration of use, median (IQR)	10 (6–14)	5 (3–8)	0.022
Antipseudomonal penicillins			
Number of patients (%)	25 (28)	1 (1.1)	<0.001
Duration of use, median (IQR)	7 (3–10)	5 (5–5)	0.74
Cephalosporins			
Number of patients (%)	2 (2.3)	2 (2.1)	1
Duration of use, median (IQR)	14 (12–15)	11 (10–12)	0.41
Carbapenems			
Number of patients (%)	2 (2.3)	0 (0.0)	0.16
Duration of use, median (IQR)	11 (9–13)	0 (0–0)	0.74
Quinolones			
Number of patients (%)	4 (4.6)	0 (0.0)	0.046
Duration of use, median (IQR)	9 (7–11)	0 (0–0)	0.022
Aminoglycosides			
Number of patients (%)	6 (6.8)	0 (0.0)	0.031
Duration of use, median (IQR)	14 (11–35)	0 (0–0)	1
Anti–MRSA agent (VCM)			
Number of patients (%)	33 (38)	74 (79)	<0.001
Duration of use, median (IQR)	4 (2–11)	12 (7–16)	<0.001
Anti–MRSA agents (DAP, LZD)			
Number of patients (%)	13 (15)	28 (30)	0.028
Duration of use, median (IQR)	5 (4–15)	4 (3–8)	0.12
Time to antibiotic therapy against enterococci (days), median (IQR)	0 (0–1)	1 (0–1)	0.049
Total duration of antibiotic therapy (days), median (IQR)	14 (8–19.3)	13 (8–17)	0.99
Daily antimicrobial cost ($), median (IQR)	23 (13–34)	34 (22–58)	<0.001
Vancomycin median serum trough concentrations (≥20 mg/L), n (%)	5 (5.7)	19 (20)	0.007
AKI after the onset of bacteremia, n (%)	7 (8.0)	20 (21)	0.02
Hospital length after the onset of bacteremia until discharge (days), median (IQR)	33 (14.8–69.5)	29 (15.3–58.5)	0.34

IQR: interquartile range, MRSA: methicillin-resistant *Staphylococcus aureus*, VCM: vancomycin, DAP: daptomycin, LZD: linezolid, AKI: acute kidney injury.

**Table 4:** Risk factors that were associated with 30-day mortality due to enterococcal bacteremia.

	Survived (n = 141)	Died (n = 41)	*p*	Adjusted OR (95% CI)	*p*
Admission to intensive care unit, n (%)	32 (23)	22 (54)	<0.001	1.65 (0.61–4.47)	0.33
Comorbidities at bacteremia, n (%)					
Chronic renal failure	52 (37)	18 (44)	0.38		
Dialysis	16 (11)	3 (7.3)	0.74		
Diabetes mellitus	27 (19)	9 (22)	0.46		
Cardiovascular disease	19 (14)	6 (15)	0.54		
Cardiac valve replacement	11 (7.8)	8 (20)	0.089		
Source of infections, n (%)					
Central venous catheter	34 (25)	7 (17)	0.003		
Cholecystocholangitis	29 (21)	9 (22)	1		
Unknown	24 (17)	16 (39)	0.014	2.79 (1.14–6.85)	0.025
Hospital stay before the onset of bacteremia (days), median (IQR)	25 (10–55)	39 (14–80)	0.16		
qSOFA score ≥ 2 at bacteremia, n (%)	33 (23)	23 (56)	<0.001	2.96 (1.15–7.62)	0.024
Previous immunosuppression (within 30 days), n (%)					
Immunosuppressive treatment	5 (3.6)	6 (15)	0.024	2.31 (0.46–11.5)	0.31
Corticosteroid treatment	24 (17)	15 (37)	0.014	2.84 (1.08–7.46)	0.034
Chemotherapy	16 (11)	2 (4.9)	0.36		
Acute kidney injury after the onset of bacteremia, n (%)	15 (11)	12 (29)	<0.001	4.51 (1.61–12.7)	0.004
*E. faecium* bacteremia, n (%)	67 (48)	27 (66)	0.059		
Antibiotic susceptibility, n (%)					
Ampicillin	57 (40)	24 (59)	0.11		
Imipenem	67 (48)	27 (66)	0.059		
Levofloxacin	65 (46)	26 (63)	0.076		

IQR: interquartile range, qSOFA: quick Sequential Organ Failure Assessment.

## Data Availability

Data sharing is not applicable to this article.
